# Impella RP Use in Refractory Cardiogenic Shock in a Patient Presenting With Acute Right Coronary Artery Occlusion: A Case Report

**DOI:** 10.7759/cureus.43072

**Published:** 2023-08-07

**Authors:** Mohammad Abdel Jawad, Abdullah Abu Kar, Andre Saad, Ali Elkharbotly, Zaher Fanari

**Affiliations:** 1 Internal Medicine, Ascension Via Christi St. Francis, Wichita, USA; 2 Hospital Medicine, University of California San Francisco, San Francisco, USA; 3 Cardiology, Ascension Via Christi St. Francis, Wichita, USA; 4 Cardiology, University of California San Francisco, Fresno, USA

**Keywords:** device therapy in heart failure, biventricular impella, right ventricular failure, right ventricular infarction, right sided cardiogenic shock

## Abstract

It is common for patients with inferior myocardial infarction to experience right ventricular infarction, occurring in half of the patients with inferior myocardial infarction. Right ventricular failure due to acute right myocardial infarction is often associated with a worse prognosis. In this case, we report a patient with acute chest pain due to acute right coronary artery occlusion status post placement of multiple stents in the right coronary artery. Unfortunately, he developed refractory cardiogenic shock requiring biventricular assist device placement.

## Introduction

Acute right ventricular failure (RVF) is associated with significant morbidity and mortality [1]. Causes of RVF include coronary artery disease, myocarditis, right-sided valvular insufficiency, acute pulmonary embolism, and the presence of a left ventricle assist device [2]. Right ventricular infarction (RVI) can lead to impaired right ventricle contractility. Management of RVF begins with medical treatments, which include optimizing right ventricle preload, treating the underlying cause (coronary revascularization for acute coronary syndrome, anticoagulation, thrombolytic or embolectomy for pulmonary embolism, or anti-inflammatory agents for myocarditis), and inotropic therapy to improve cardiac output. For RVF refractory to medical management, invasive treatment modalities should be utilized. Here, we present a case of a patient with acute cardiogenic shock due to right ventricular infarction that was refractory to medical management. He underwent biventricular assist device placement as a bridge to recovery.

## Case presentation

**Figure 1:**
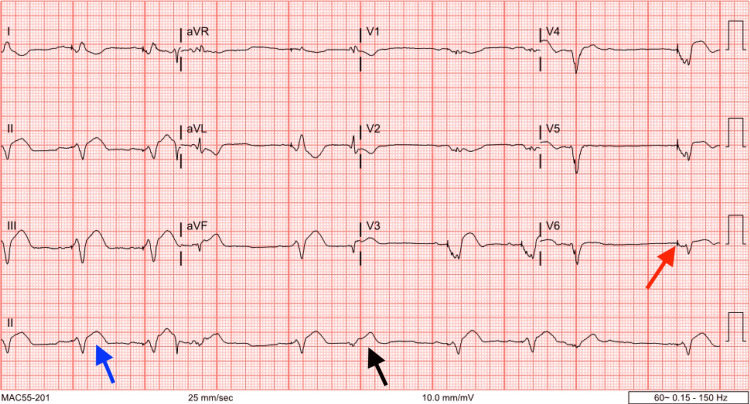
Patient’s initial electrocardiogram on presentation to the hospital Electrocardiogram on presentation showing sinus rhythm with a ventricular paced rhythm (red arrow pointing at ventricular pacing spike) and a wide bizarre paced QRS complex (blue arrow). Occasional premature ventricular complexes can be seen (black arrow).

A 72-year-old man with a history of coronary artery disease, paroxysmal atrial fibrillation, hypertension, hyperlipidemia, ischemic cardiomyopathy, and heart failure with reduced ejection fraction status post automatic implantable cardioverter defibrillator placement. The patient presented to the emergency department with mid-sternal chest pain that started one hour before arrival. On physical examination, the heart rate was 63 beats per minute, respiratory rate was 18 breaths per minute, blood pressure was 112/64, and oxygen saturation was 98% while the patient was breathing ambient air. The patient appeared in distress and was diaphoretic. A cardiac examination revealed bradycardia with a regular rhythm; no murmurs or gallops were appreciated. Lung examination revealed clear breath sounds bilateral. Electrocardiogram (ECG) on admission showed sinus rhythm with a ventricular-paced rhythm, wide QRS complex, and occasional premature ventricular complexes (Figure 1). Initial Troponin was negative. While in the emergency department, the patient went into Ventricular Fibrillation (V-Fib) cardiac arrest with successful cardiopulmonary resuscitation (CPR) and a return of spontaneous circulation after 3 minutes of CPR. During CPR, the patient received 1mg of intravenous epinephrine and 300mg of intravenous Amiodarone as a bolus. The catheterization laboratory at our hospital was activated, and the patient was emergently transferred to our hospital.

**Figure 2:**
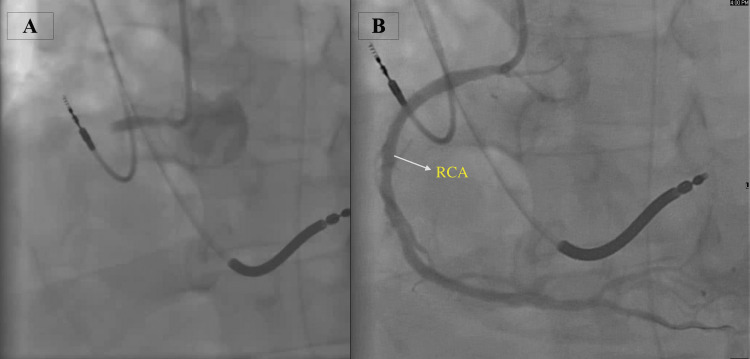
Coronary angiography. Angiography displaying the right coronary artery before (A), and then after percutaneous coronary intervention (B). RCA denotes the right coronary artery. RCA: right coronary artery

The patient arrived in the catheterization laboratory in severe cardiogenic shock. He was on three pressors (norepinephrine, epinephrine, and vasopressin). He underwent selective coronary angiography that revealed 80% ostial right coronary artery (RCA) stenosis followed by complete occlusion of the mid-RCA (Figure 2A). The patient received percutaneous coronary intervention (PCI) with the placement of a 3.5 x 12 mm Xience Sierra drug-eluting stent (Abbott, Chicago, IL) into the ostial RCA, a 3.5 x 18 mm Xience Sierra drug-eluting stent in the mid-RCA, and a 3.0 x 12 mm Xience Sierra drug-eluting stent in the distal RCA. The distal left anterior descending artery had a very focal 80% stenosis that was not amenable to PCI. Post-intervention, the perfusion in the RCA improved to a Thrombolysis in Myocardial Infarction (TIMI) flow grade 3 (Figure 2B). Subsequently, blood pressure remained extremely low, requiring three pressors. The left heart catheterization revealed a high left ventricular end-diastolic pressure measuring 49 mmHg with a left ventricular pressure of 135 mmHg. Following the National Cardiogenic Shock Initiative protocol [3], a decision was made to place a left ventricular assist device. At this point, the aortic valve was crossed with a pigtail catheter. The groin sheath was upsized for a 13-French sheath, and then an Impella CP (Abiomed, Danvers, MA) was placed into the left ventricle (LV) and secured in place. Once turned on, epinephrine infusion was weaned off, and the other two pressors were continued. The patient was transferred to the cardiac intensive care unit in critical condition. He was on a heparin drip, and his activated clotting time was closely monitored with a goal of 160-180s.

**Figure 3:**
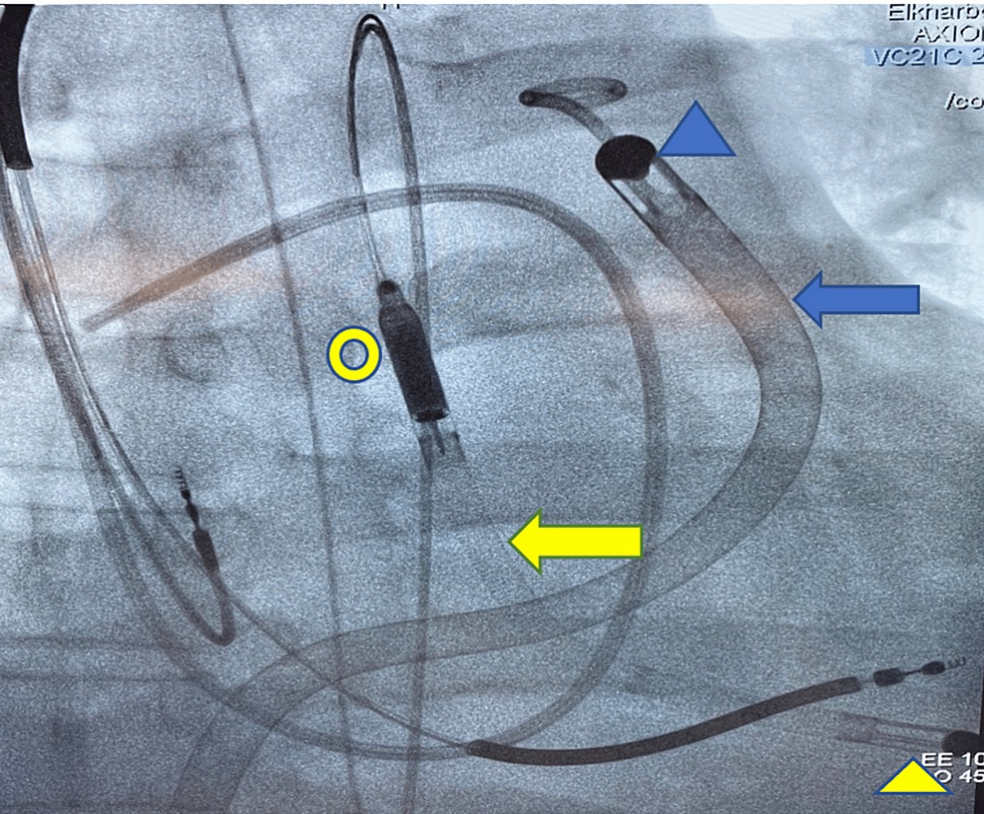
Fluoroscopic image showing biventricular Impella Fluoroscopic image showing left ventricle Impella (yellow arrow), and Impella RP (blue arrow). The left ventricle Impella has its inlet in the left ventricle (yellow arrowhead) and outlet in the ascending aorta (yellow circle), while the Impella RP has its inlet in the inferior vena cava–right atrial junction (not shown in this image) and outlet in the pulmonary artery (blue arrowhead).

The following day, the patient's condition continued to deteriorate despite fluid resuscitation, LV Impella support, increasing norepinephrine and vasopressin requirements, and starting inotropic therapy with milrinone. He developed an anuric acute kidney injury and became more acidotic, requiring renal replacement therapy. Echocardiogram revealed severely reduced RV function (Video 1). The multidisciplinary heart team discussed and agreed to place a temporary percutaneous right ventricular assist device. Right heart catheterization with hemodynamic assessment showed a pulmonary artery pulsatility index (PAPi) of 0.6, indicating severe RV dysfunction. Impella RP (Abiomed, MA) was placed through the right femoral vein access. The Impella RP was appropriately positioned and sutured in place. The left and right ventricular Impella devices were functioning well (Figure 3). On day four of admission, the patient was recovering well. The hemodynamic assessment showed his PAPi was up to 1.7, confirming RV's functional recovery. Over the next few days, the patient was weaned off the RV Impella and LV Impella with recovered renal function. Echocardiography on hospital day 6 showed normal RV systolic function (Video 2). Three weeks after discharge from the hospital, he was seen in the cardiology clinic, and he was doing well with minimal symptoms on exertion.

**Video 1:** Echocardiography on hospital day 2 showing impaired right ventricular systolic function

**Video 2:** Echocardiography on hospital day 6 showing normal right ventricle contraction

## Discussion

Right ventricular infarction involves up to half of the patients presenting with inferior myocardial infarction (MI) [4]. Compared to patients with isolated inferior MI, patients with inferior MI and concomitant RV myocardial involvement have a higher incidence of in-hospital complications, including cardiogenic shock, along with higher mortality [1]. RVI can lead to acute RVF, which should be suspected in patients with refractory cardiogenic shock. RVF is associated with substantial morbidity, mortality, and extended hospital stays [5].

Typical physical exam findings of RV dysfunction include elevated jugular venous pressure, clear lung fields, and hypotension. Echocardiography is essential in diagnosing RV failure. The Pulmonary artery pulsatility index (PAPi) is a hemodynamic index that can predict RV failure. It is calculated as follows: pulmonary artery systolic pressure - pulmonary artery diastolic pressure/right atrial pressure. A PAPI of < 1.0 in the acute inferior MI setting indicates severe RV failure [6].

Management of RVF entails maintaining adequate preload return to the heart while avoiding volume overload, reducing RV afterload using pulmonary vasodilator therapy, inotropic therapy, revascularization of obstructive coronary artery disease, and mechanical circulatory support (MCS), if needed. Different types of percutaneous RV MCS [2]; direct RV bypass devices include Impella RP and TandemHeart right ventricular assist devices. Indirect RV bypass includes veno-arterial extracorporeal membrane oxygenation. RV MCS is essential in managing patients with acute RV failure with cardiogenic shock refractory to other medical therapies.

Following positive results from the Recover Right trial [7], Impella RP received US Food and Drug Administration approval in 2017 for treating: acute right heart failure following myocardial infarction, heart transplant, left ventricular assist device placement, or open-heart surgery. Impella RP is a percutaneous microaxial flow right ventricular pump placed with its inlet in the inferior vena cava-right atrial junction. Venous blood is delivered from the inlet area through its cannula into the outlet area of the pulmonary artery. A few of the contraindications for using this device include tricuspid or pulmonic valve severe stenosis or regurgitation and disorders of the pulmonary artery.

We presented a case of a patient presenting with chest pain secondary to acute RCA occlusion. His clinical condition continued to decompensate while on pressor and inotropic support, an inserted LV Impella, and following PCI of the culprit lesion. RV MCS was our patient's appropriate next management intervention, which was successful as a bridge to recovery.

## Conclusions

Acute myocardial infarction can lead to right ventricular failure, which may be further complicated by cardiogenic shock. In cases where patients experience refractory cardiogenic shock due to inferior myocardial infarction or right coronary artery occlusion, it is essential to consider using a right ventricular mechanical circulatory support device strongly. This approach has the potential to significantly improve the patient's chances of recovery and survival, especially when conventional treatments alone may not be sufficient to address the severity of the condition.
